# Selection and validation of reference genes for RT-qPCR-based analyses of *Anastatus japonicus* Ashmead (Hymenoptera: Helicopteridae)

**DOI:** 10.3389/fphys.2022.1046204

**Published:** 2022-10-19

**Authors:** Zixin Liu, Junjiang Xiao, Yue Xia, Qifeng Wu, Can Zhao, Dunsong Li

**Affiliations:** ^1^ Key Laboratory of Green Prevention and Control on Fruits and Vegetables in South China Ministry of Agriculture and Rural Affairs, Guangdong Provincial Key Laboratory of High Technology for Plant Protection, Institute of Plant Protection, Guangdong Academy of Agricultural Sciences, Guangzhou, China; ^2^ College of Plant Protection, South China Agricultural University, Guangzhou, China

**Keywords:** *Anastatus japonicus*, RT-qPCR, reference gene, diapause, normalization

## Abstract

RT-qPCR remains a vital approach for molecular biology studies aimed at quantifying gene expression in a range of physiological or pathological settings. However, the use of appropriate reference genes is essential to attain meaningful RT-qPCR results. *Anastatus japonicus* Ashmead (Hymenoptera: Helicopteridae) is an important egg parasitoid wasp and natural enemy of fruit bugs and forest caterpillars. While recent transcriptomic studies have analyzed gene expression profiles in *A. japonicus* specimens, offering a robust foundation for functional research focused on this parasitoid, no validated *A. japonicus* reference genes have yet been established, hampering further research efforts. Accordingly, this study sought to address this issue by screening for the most stable internal reference genes in *A. japonicus* samples to permit reliable RT-qPCR analyses. The utility of eight candidate reference genes (ACTIN, TATA, GAPDH, TUB, RPL13, RPS6, EF1α, RPS3a) was assessed under four different conditions by comparing developmental stages (larvae, pupae, adults), tissues (abdomen, chest, head), sex (male or female adults), or diapause states (diapause induction for 25, 35, 45, or 55 days, or diapause termination). RefFinder was used to calculate gene stability based on the integration of four algorithms (BestKeeper, Normfinder, geNorm, and ΔCt method) to determine the optimal RT-qPCR reference gene. Based on this approach, RPS6 and RPL13 were found to be the most reliable reference genes when assessing different stages of development, while ACTIN and EF1α were optimal when comparing adults of different sexes, RPL13 and EF1α were optimal when analyzing different tissues, and TATA and ACTIN were optimal for different diapause states. These results provide a valuable foundation for future RT-qPCR analyses of *A. japonicus* gene expression and function under a range of experimental conditions.

## Introduction

*Anastatus japonicus* Ashmead (Hymenoptera: Helicopteridae) is an important egg parasitoid wasp that preys on a range of fruit bugs and forest caterpillars including *Halyomorpha halys* Stal, *Tessaratoma papillosa* Drury, and *Riptortus pedestris* Fabricius (Zhao et al., 2019a; Chen et al., 2019; Mi et al., 2020; Chen et al., 2021; Zhao et al., 2021; Wei et al., 2022). In commercial settings, *A. japonicus* is used as a form of biological control agent that is reared within *Antheraea pernyi* Guerin-Meneville eggs (Lepidoptera: Saturniidae) (Zhao et al., 2021). Moderate springtime temperatures induce diapause in *A. japonicus,* resulting in delayed adult emergence and interfering with the optimal timing for pest control, thus limiting the biocontrol value of this wasp species (Zhao et al., 2019b; Zhao et al., 2021). Relative to *A. japonicus* individuals not in diapause, those in diapause exhibit higher survival rates following storage at 10°C for 180 days (Zhao et al., 2021)*.* Our group recently conducted transcriptomic sequencing for non-diapause and diapause *A. japonicus* individuals (unpublished data), revealing the presence of many genes associated with energy metabolism, development, and oxidation-reduction reactions. Further studies of these genes, however, will be vital to clarify the molecular basis for diapause in *A. japonicus*. No published studies to date have sought to analyze *A. japonicus* gene expression profiles, highlighting the need for additional foundational research to support further study of this potentially economically valuable biocontrol species.

Quantitative real-time PCR (RT-qPCR) analyses offer a rapid, sensitive, efficient, accurate, and reproducible means of measuring gene expression under a range of analytical conditions while also permitting single nucleotide polymorphism and restriction fragment length polymorphism analyses (Wang et al., 2019). However, the reliability of RT-qPCR analyses is strongly dependent on factors including the quality and yield of utilized templates, the amplification efficiency of the selected primers, and underlying biological signal (Jiang et al., 2015; Shu et al., 2018; Hu et al., 2019). In the process of RT-qPCR, in order to ensure the accuracy of quantitative results and achieve normalization and correction of different samples, the introduction of internal reference genes is indispensable (Lu et al., 2013; Taylor et al., 2016). Housekeeping genes often used for this purpose across species and treatment conditions include 18S rRNA and the genes encoding tubulin, ACTIN, and glyceraldehyde-3-phosphate dehydrogenase (GAPDH). However, a growing number of studies have revealed that these commonly utilized housekeeping genes do not exhibit stable expression patterns across all experimental conditions, potentially leading to skewed, inaccurate data. It is thus vital that the most appropriate reference genes for particular RT-qPCR applications be selected in a systematic manner to maximize the reliability and accuracy of associated experimental results.

Here, eight commonly utilized reference genes were evaluated to establish their value when normalizing RT-qPCR data for *A. japonicus* specimens. These genes included ribosomal protein S3a (RPS3a), GAPDH, ACTIN, elongation factor 1α (EF1α), TATA-box binding protein (TATA), ribosomal protein S6 (RPS6), β -tubulin (TUB), and ribosomal protein L13 (RPL13). All of which have been widely used as reference genes in different organisms because they are considered to have a uniform expression.The EF1α and TUB were thought to be good reference genes for most animals (Zhao et al., 2019a; Wang et al., 2021). However, the absence of poly-A in rRNA makes it impossible to isolate it using oligo (dT)-based techniques, which is a significant disadvantage when employing rRNA as a normalizer in RT-qPCR experiments (Xue et al., 2010). Thus, we focused on genes transcribed by RNA polymerase II whose transcripts can be isolated using oligo (dT), like the genes linked to GAPDH, ACTIN and ribosomal proteins (e.g., RPS3a, RPS6, and RPL13) (Galetto et al., 2013; Xu et al., 2021; Li et al., 2021; Shen et al., 2022). And the TATA gene is required for initiating the RNA polymerase (I, II, and III)-mediated transcription of genes with promoters with or without a TATA box (Pugh, 2000). So, Those eight reference genes were chosen. Expression dynamics for these candidate reference genes were compared with RefFinder, which integrates results from four different algorithms (BestKeeper, NormFinder, geNorm, and the ΔCt method). The stability of these genes was compared across three biotic conditions (sex, tissue type, or developmental stage) and one abiotic condition (diapause state). The goal of this study was to define the *A. japonicus* reference genes that are most appropriate for use across a range of experimental conditions. These data may offer value as a foundation for future more reliably RT-qPCR-based studies of *A. japonicus.*

## Materials and methods

### Insect rearing

*A. japonicus* Ashmead specimens were obtained from the Institute of Plant Protection of the Chinese Academy of Agricultural Sciences, Ministry of Agriculture and Rural Affairs-CABI Biosafety Joint Laboratory. The initial *A. japonicus* colony was established from brown marmorated stink bug (*Halyomorpha halys* Stal) eggs that had been parasitized under field conditions and were collected from the suburbs of Beijing. *A. japonicus* colonies were maintained for 1 year under laboratory conditions (24°C, 16 h light/8 h dark cycle) using *Antheraea pernyi* eggs. After newly emerging, a solution of 10% honey and water was used to feed *A. japonicus* individuals in a 32 cm × 25 cm × 9 cm plastic box under these same laboratory conditions.

### Sample preparation

#### Biotic factors

All *A. japonicus* developmental stages were analyzed for this study (including mature larvae (*n* = 8), pupae (*n* = 10), and male (*n* = 20) and female (*n* = 10) adult individuals sampled on day 1 of the corresponding stage. In addition, samples from the head, chest, and abdomen of ∼40 adult female individuals were collected for analysis.

#### Abiotic factors

To induce diapause, *Antheraea pernyi* eggs were parasitized by *A. japonicus* for 2 days under standard conditions (24°C ± 0.5°C, 70% relative humidity, 16L:8D photoperiod), after which they were transferred to diapause-inducing conditions (17°C, 10L:14D photoperiod) for 25, 35, 45, or 55 days. Diapause was terminated after 55 days by transferring *A. japonicus* individuals back to normal developmental conditions (24°C, 16L:8D) for 12 days.

Each treatment was repeated in triplicate (*n* = 15 individuals/treatment). Samples were stored in 1.5 ml RNA-free tubes, snap-frozen with liquid nitrogen, and stored at −80°C.

#### Total RNA extraction and cDNA synthesis

The MiniBEST Universal RNA Extraction Kit (TaKaRa, China) was used to isolate RNA based on provided directions. RNA concentration levels were measured with a NanoDrop ND-2000C instrument (Thermo Fisher Scientific, MA, United States). Total RNA was prepared in a 50–100 µl volume of ddH_2_O at the following concentrations (mean ± SEM): 651.8 ± 35.6 ng/μl for mature larvae, 1,013.7 ± 180.3 ng/μl for pupae, 317.9 ± 162.8 ng/μl for female adults, 275.9 ± 119.8 ng/μl for male adults, 335.3 ± 50.7 ng/μl for head samples, 429.8 ± 86.5 ng/μl for chest samples, 596.6 ± 233.2 ng/μl for abdomen samples, 1,180.9 ± 74.7, 328.1 ± 184.9, 278.1 ± 80.4 and 266.9 ± 83.5 ng/μl for 25, 35, 45, and 55 days under diapause-inducing conditions, and 258.9 ± 30.7 ng/μl for diapause termination.

All samples exhibited OD260/280 values from 1.9 to 2.1. First-strand cDNA synthesis was performed using a PrimeScript RT kit containing gDNA Eraser (Perfect Real Time, TaKaRa, China). All cDNA samples were diluted 10-fold for RT-qPCR analyses.

#### Primer design

For this study, RT-qPCR was used to examine the expression of eight commonly utilized reference gene candidates (Table 1), using primers designed with the PrimerQuest Tool (https://sg.idtdna.com/PrimerQuest/Home/Index) based on sequences derived from our recent transcriptomic analysis of *A. japonicus* (unpublished data).

**TABLE 1:** Primers and amplicon characteristics for the selected reference gene candidates.

Gene	Genebank numbers	Primer sequences (5–3)	Length (bp)	Efficiency (%)	R^2^	Linear regression
EF1a	OP561762	F:GAGCAGTATGGCAATCGAGTA R:CAAAGAGCAACCCACCAAAG	114	102.06	0.9985	y = −3.2736x + 25.342
TATA	OP561763	F:CCTAGAACGACGGCACTTATT R:CTTGGCTGGAAACCCTAGTT	129	99	0.9937	y = −3.3461x + 34.548
TUB	OP561764	F:GAGCCATCCTTGTGGATCTT R:TGACCTTTGGCCCAGTTATT	128	107.67	0.9909	y = −3.1508x + 30.149
GAPDH	OP561765	F:CATCCCGTGTCATCGATCTTAT R:TGCCACGTCTCTTCCTTTC	119	102.67	0.9994	y = −3.2596x + 27.254
RPS6	OP561766	F:CCCTAAGCGACGAGGTATATTG R:CGCAAATCAGTTCGTGGATG	123	105.76	0.9984	y = −3.1913x + 26.08
RPS3a	OP561767	F:GGCGATAGCATCAGGTAACA R:GCACAGGTTAGGAGCATTAGA	108	105.43	0.9982	y = −3.1984x + 25.744
RPL13	OP561768	F:TCAACCAGCCCGCAAATA R:CTTTGCCTGCACGAACTTTAG	140	103.68	0.999	y = −3.2368x + 26.008
ACTIN	OP561769	F:GGCTGTCCTGTCGCTTTAT R:CGTAGGATGGCATGAGGTAAA	120	107.1	0.9942	y = −3.1627x + 26.965

### Quantitative real-time PCR analysis

TB Green^®^ Premix Ex Taq™ II (Tli RNaseH Plus) (Takara) and a CFX Connect Real-Time Instrument (Bio-Rad, CA, United States) were used for all RT-qPCR analyses, which were conducted in a total volume of 50 µl containing 25 µl of TB green, 2.5 µl of each primer (F + R; 100 µM), 2.5 µl of diluted cDNA, and 17.5 µl of RNase-free ddH_2_O. Amplification settings were as follows: 95°C for 30 s; 39 cycles of 95°C for 5 s and 55°C for 30 s; Melting curves for the product were determined by raising the temperature from 55°C to 90°C in sequential steps of 0.5°C for 1 s. For each primer, standard curves were produced using a two-fold dilution series of cDNA as the template according to the linear regression model. All analyses were conducted using three biological and three technical replicates. RT-qPCR amplification efficiency (E) were measured as follows: E = [10 (−1/slope)−1] × 100 (Pfaffl, 2001).

### Data analysis

The number of cycle (Ct) values required for amplification levels to reach a fixed exponential phase threshold (set to 500 for all genes) during PCR analyses was used to compute gene expression. RefFinder (http://blooge.cn/RefFinder/), which is a tool that integrates results from the NormFinder (Andersen et al., 2004), geNorm (Vandesompele et al., 2002), ΔCt method (Silver, et al., 2006) and BestKeeper (Pfaffl et al., 2004)analytical tools, was utilized to assess the stability of these different *A. japonicus* reference gene candidates. In addition, the geNorm tool was utilized to establish the optimal number of reference genes based on V_n_/V_n+1_ values, with a V_n_/V_n+1_ > 0.15 necessitating the addition of an additional reference gene whereas no further reference genes are required when V_n_/V_n+1_ < 0.15, with 0.15 thus serving as the threshold for additional reference genes gene incorporation (Vandesompele et al., 2002; Liang et al., 2014).

## Results

### Amplification efficiencies

**FIGURE 1:**
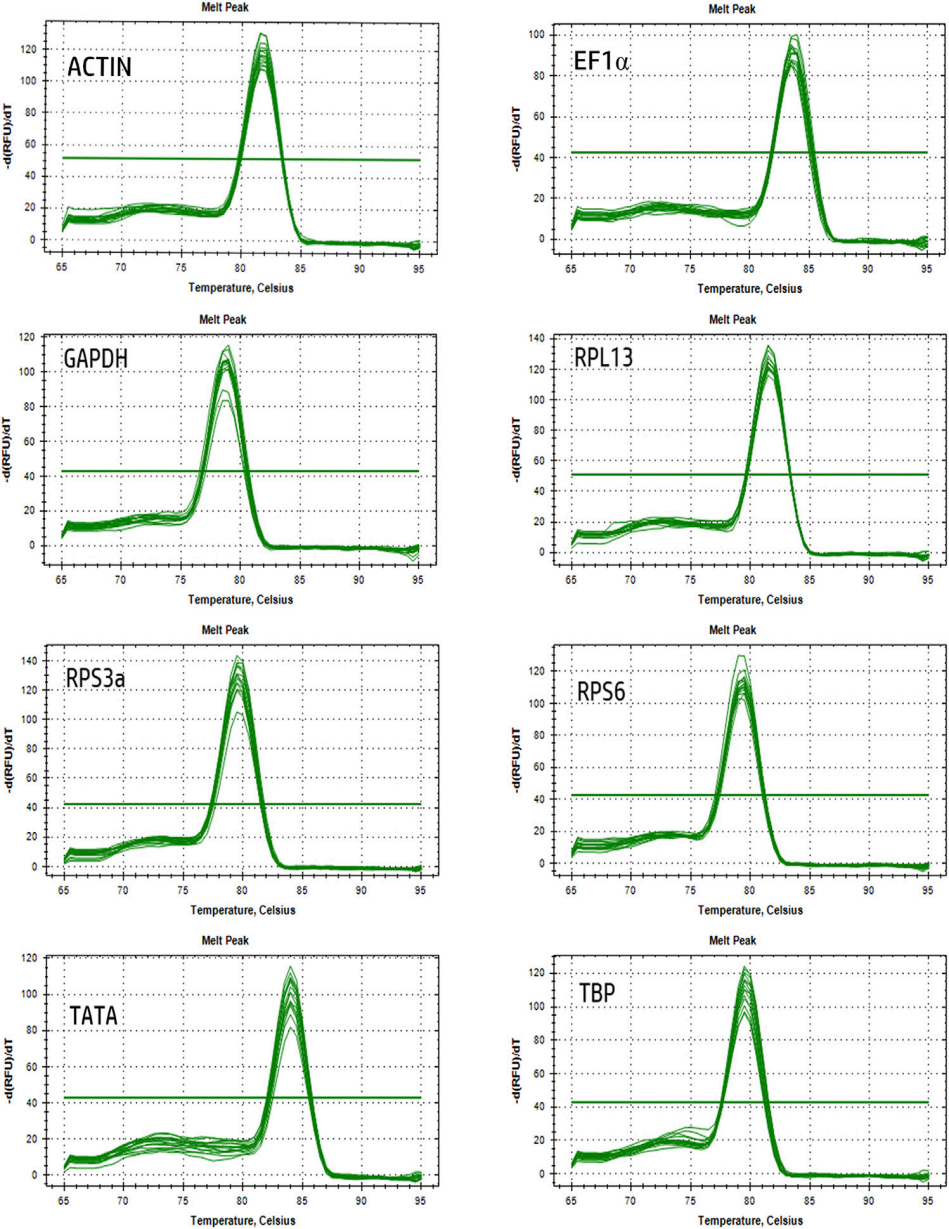
Melting curves of the eight candidate reference genes in *A. japonicus*.

All eight of the tested reference gene candidates were expressed at detectable levels in the analyzed *A. japonicus* samples, with 1% agarose gel electrophoresis results for each transcript exhibiting a single amplicon of the expected size. Amplification efficiency for each gene was estimated using two-point standard curves with known RNA concentrations, while melt curves were used to confirm the specificity of these amplification results based on the presence of a single peak without any evidence of primer-dimer peaks (Figure 1). Amplification efficiency (E) values for these genes ranged from 99% to 107.67%, with an R^2^ > 0.9909 (Table 1).

### Expression profiles of candidate reference genes

**FIGURE 2:**
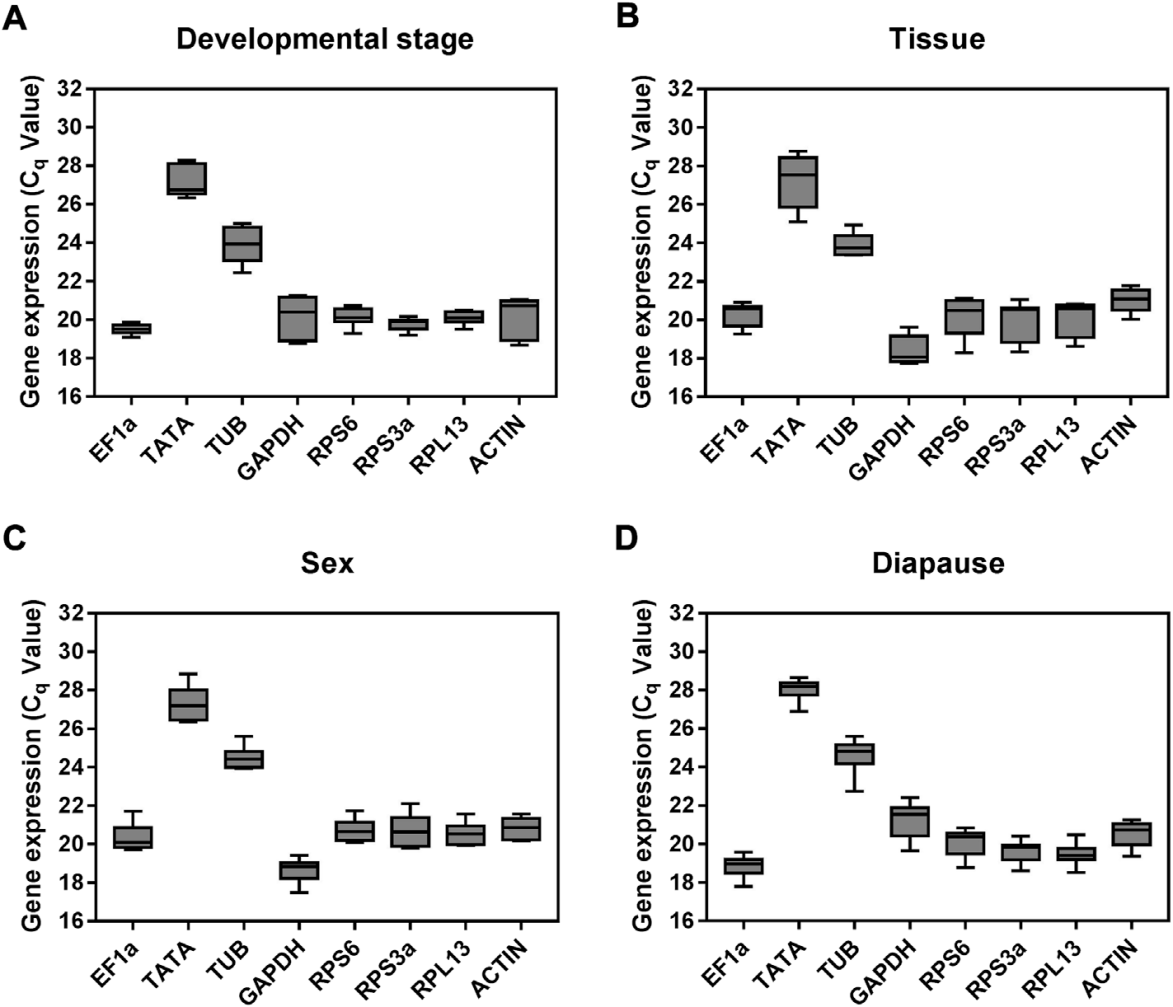
Expression profiles of the eight candidate reference genes in all four experiments for *A. japonicus*. The expression levels of the reference genes are shown in terms of the Cq-value for each experimental condition. **(A)** Development stage, **(B)** Tissue, **(C)** Sex, **(D)** Diapause treatment.

Expression levels for these candidate reference genes varied widely across the tested experimental conditions with respect to the measured Ct values (Figure 2), ranging from a Ct value of 17.80 for GAPDH to 28.69 for TATA. Mean respective Ct values for TATA and TUB were 27.65 and 24.28, with these values being notably higher than those for other tested genes. Moderate expression was observed for the other candidate reference genes included in this study, with respective mean Ct values for EF1α, GAPDH, RPS6, RPS3a, RPL13, and ACTIN of 19.53, 20.07, 20.40, 19.88, 19.90, and 20.56.

### Analyses of the stability of *A. japonicus* reference genes under different experimental conditions and optimum number of genes for normalization

#### Developmental stage analysis

**FIGURE 3:**
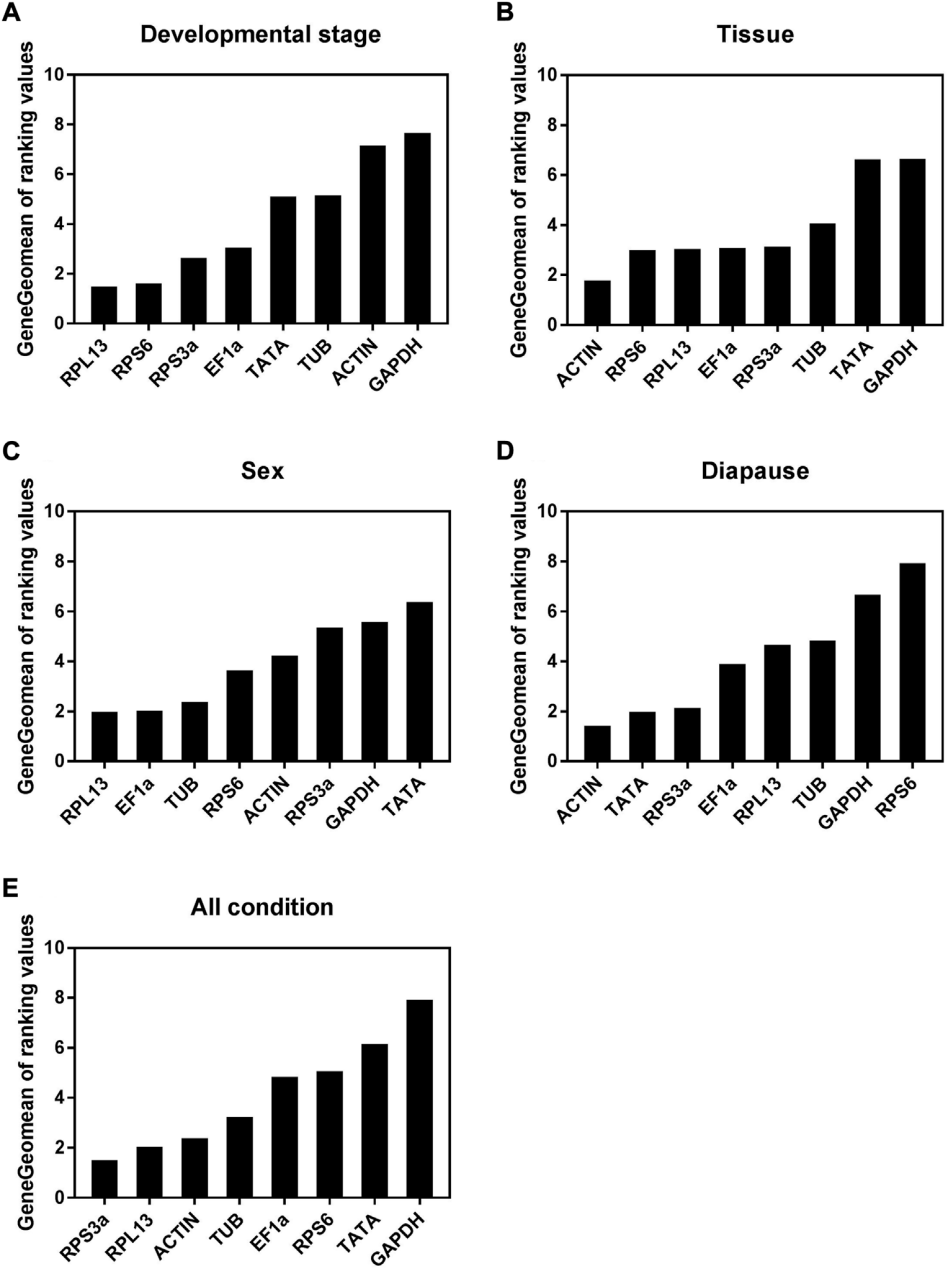
Stability of the eight candidate reference gene expressions in *A. japonicus* under different treatment conditions analyzed using RefFinder. **(A)** Development stage, **(B)** Tissue, **(C)** Sex, **(D)** Diapause treatment, **(E)**All condition.

**FIGURE 4:**
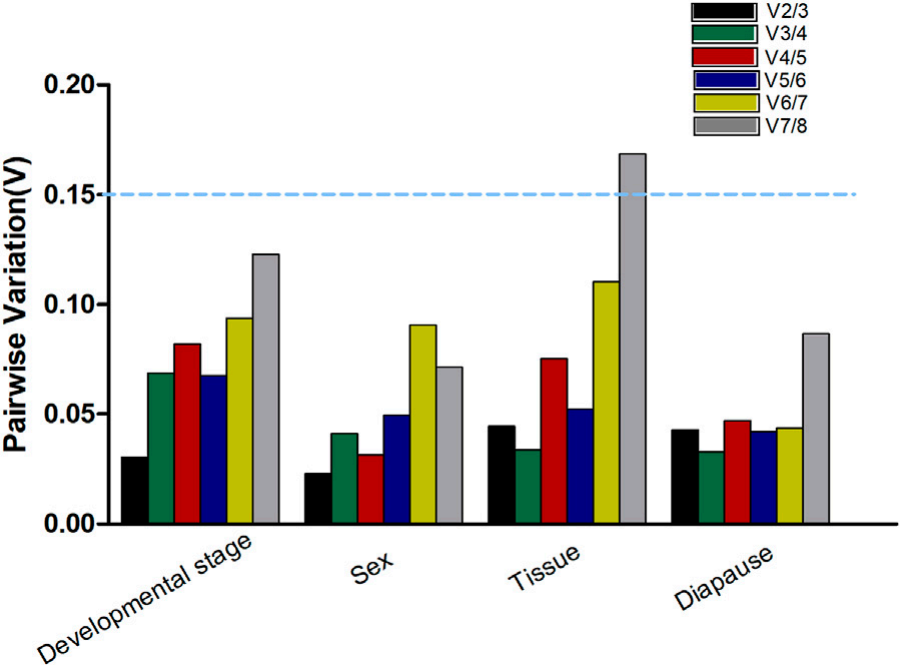
Optimum number of reference genes required for accurate normalization of gene expression. Pairwise variation (V) values in the four groups using geNorm.

When analyzing *A. japonicus* samples collected during different stages of development, the geNorm, NormFinder, and ΔCt analytical approaches revealed RPS6 and RPL13 as the most stable reference genes (Table 2). In contrast, BestKeeper analyses suggested that Ef1α and RPS3a were instead the most stable, with ACTIN and GAPDH as the least stable genes. Per RefFinder, the overall rank order for the stability of these reference genes was: RPL13, RPS6, RPS3a, EF1α, TATA, TUB, ACTIN, GAPDH (Figure 3A). In geNorm analyses, the pairwise V2/3 value was below the established 0.15 cut-off threshold (Figure 4). Overall, RefFinder thus suggested that RPL13 and RPS6 are the most appropriate target genes when analyzing *A. japonicus* samples across different stages of developmen*t* (Table 2).

**TABLE 2:** Overall rankings for *A. japonicus* reference genes.

Conditions	References gene	geNorm		NormFinder		BestKeeper		ΔCt		Recommendation
		Stability	Rank	Stability	Rank	Stability	Rank	Stability	Rank	
Developmental stage	EF1α	0.256	4	0.622	6	0.214	1	0.828	4	RPL13
	TATA	0.576	6	0.545	4	0.719	5	0.862	6	
	TUB	0.463	5	0.582	5	0.757	6	0.848	5	
	GAPDH	0.854	8	1.320	8	0.968	7	1.378	8	
	RPS6	0.107	1	0.054	1	0.313	4	0.593	1	RPS6
	RPS3a	0.129	3	0.210	3	0.22	2	0.631	3	
	RPL13	0.107	1	0.054	1	0.264	3	0.602	2	
	ACTIN	0.680	7	0.996	7	0.973	8	1.093	7	
Tissue	EF1α	0.367	5	0.114	1	0.5465	2	0.751	5	ACTIN
	TATA	0.469	6	0.974	7	1.218	8	1.063	6	
	TUB	0.669	7	0.841	6	0.435	1	1.158	7	
	GAPDH	0.972	8	1.85	8	0.693	4	1.882	8	
	RPS6	0.139	3	0.385	3	0.894	5	0.721	2	RPS6
	RPS3a	0.039	1	0.500	5	0.944	7	0.742	3	
	RPL13	0.039	1	0.488	4	0.929	6	0.743	4	
	ACTIN	0.318	4	0.114	1	0.554	3	0.716	1	
Sex	EF1α	0.001	1	0	1	0.638	5	0.422	1	RPL13
	TATA	0.305	6	0.660	6	0.941	8	0.685	5	
	TUB	0.081	3	0.038	3	0.553	4	0.422	1	
	GAPDH	0.593	8	0.862	8	0.025	2	0.878	8	
	RPS6	0.119	4	0.038	3	0.499	3	0.443	4	EF1α
	RPS3a	0.248	5	0.581	5	0.896	7	0.629	6	
	RPL13	0.001	1	0	1	0.639	6	0.422	1	
	ACTIN	0.498	7	0.818	7	0	1	0.846	7	
Diapause	EF1α	0.285	5	0.343	5	0.419	2	0.497	5	ACTIN
	TATA	0.196	3	0.214	2	0.381	1	0.437	3	
	TUB	0.349	6	0.227	4	0.673	6	0.482	4	
	GAPDH	0.393	7	0.376	6	0.724	7	0.547	7	
	RPS6	0.531	8	0.91	8	0.837	8	0.947	8	TATA
	RPS3a	0.117	1	0.219	3	0.451	4	0.416	2	
	RPL13	0.251	4	0.432	7	0.427	3	0.526	6	
	ACTIN	0.117	1	0.129	1	0.487	5	0.396	1	
All conditions	EF1α	0.426	3	0.89	7	0.693	4	1.089	7	RPS3a
	TATA	0.829	7	0.598	6	0.769	6	1.027	6	
	TUB	0.786	6	0.462	1	0.753	5	0.975	4	
	GAPDH	1.087	8	1.781	8	1.303	8	1.861	8	
	RPS6	0.722	5	0.576	4	0.779	7	1.004	5	RPL13
	RPS3a	0.24	1	0.5	2	0.662	3	0.878	1	
	RPL13	0.24	1	0.576	5	0.657	2	0.903	2	
	ACTIN	0.572	4	0.56	3	0.631	1	0.957	3	

#### Tissue-based analyses

Different reference genes were found to be the most stable when performing tissue-based analyses of *A. japonicus* samples using these different software programs (Table 2). Specifically, RPS3a and RPL13 were identified as the most stable reference genes in these different tissue types according to geNorm, while EF1α and ACTIN were the most stable according to NormFinder, EF1α and TUB were the most stable according to BestKeeper, and the ΔCt identified ACTIN and RPS6 as the most stable reference gene candidates. GAPDH was identified as being the least stable reference gene in the geNorm, NormFinder, and BestKeeper analyses. Per RefFinder, the overall rank order for the stability of these reference genes was: ACTIN, RPS6, RPL13, EF1α, RPS3a, TUB, TATA, GAPDH (Figure 3B). The pairwise V2/V3 value identified based on geNorm data was below the established 0.15 cut-off threshold, although the V7/8 value was above this threshold (Figure 4). Overall, the RefFinder analyses indicated that ACTIN and RPS6 are the most appropriate reference genes for use when normalizing *A. japonicus* gene expression across different tissue types (Table 2).

#### Sex-based analyses

When comparing gene expression results for male and female *A. japonicus* specimens, the geNorm, NormFinder, and ΔCt methods identified EF1α and RPL13 as the most stable candidate reference genes (Table 2). While BestKeeper found ACTIN and GAPDH to be the most stable genes, the other three analytical tools suggested that GAPDH was instead the least stable reference gene. Per RefFinder, the overall rank order for the stability of these reference genes was: RPL13, EF1α, TUB, RPS6, ACTIN, RPS3a, GAPDH, TATA (Figure 3C). In the geNorm analysis, the V2/V3 pairwise value was below the established 0.15 cut-off threshold (Figure 4). Overall, these RefFinder results suggest that RPL13 and EF1α are the most appropriate reference genes for use when normalizing gene expression levels in *A. japonicus* specimens of different sexes (Table 2).

### Diapause treatment analyses

When evaluating the different tested diapause treatments, ACTIN and TATA were identified by geNorm analyses as the most stable reference genes, whereas RPS6 was the least stable. The ΔCt and NormFinder analyses also yielded similar results (Table 2). BestKeeper, in contrast, identified RPL13 and EF1α as being the most stable. Per RefFinder, the overall rank order for the stability of these reference genes was: TATA, ACTIN, RPL13, RPS3a, EF1α, TUB, GAPDH, and RPS6 (Figure 3D). All pairwise variation values were under the 0.15 cut-off threshold in geNorm analyses (Figure 4). According to the RefFinder results, ACTIN and TATA were thus the most reliable reference genes when normalizing target gene expression over a range of diapause treatment conditions.

### All conditions

When evaluating all treatment conditions, the ΔCt and geNorm analyses revealed that RPL13 and RPS3a were the most stable reference genes (Table 2). From most to least stable, the RefFinder analysis ranked the stability of these candidate *A. japonicus* reference genes as follows: RPS3a, RPL13, ACTIN, TUB, EF1α, RPS6, TATA, GADPH (Figure 3E). In geNorm analyses, all pairwise variation values were under the selected cut-off value of 0.15 (Figure 4). Overall, this RefFinder analysis indicated that RPS3a and RPL13 are the most appropriate genes for internal reference use when normalizing *A. japonicus* gene expression in RT-qPCR analyses (Table 2).

### Reference gene validation

**FIGURE 5:**
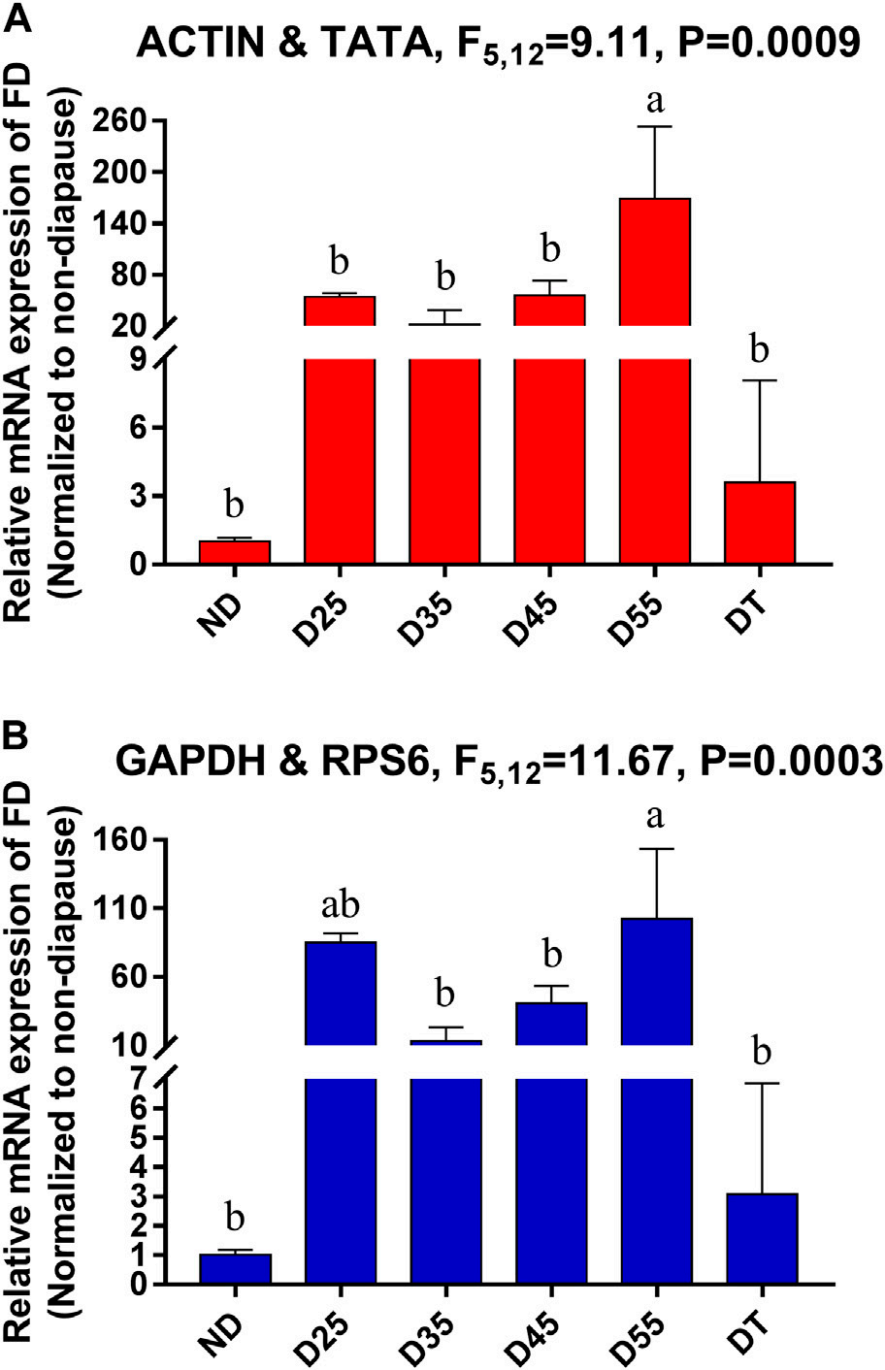
Relative gene expression of *FD* in different tissues of *A. japonicus*. The relative abundance of *FD* in the ND, D25, D35, D45, D55 and DT were normalized to the best stable (A, *ACTIN* and *TATA*) and least stable (B, *GAPDH* and *RPS6*) reference genes, respectively. ND, non-diapause mature larvae; D25, D35, D45, D55, diapausing mature larvae which incubated in diapause-inducing conditions for 25, 35, 45, 55 days; DT, diapause termination mature larvae. The values are means + SE. Different letters indicate significant differences in gene expression among different tissues of *A. japonicus* (*p* < 0.05).

*FD* (Farnesol dehydrogenase) is an important enzyme in the diapause process of insects and can oxidize farnesol to farnesol (the precursor of JH) (Mayoral et al., 2009). Relative *FD* expression levels were next used for the validation of these reference genes among the tested diapause treatment conditions. In general, expression patterns were similar across conditions, although these expression levels were 54- and 85-fold higher on diapause day 25 relative to non-diapause treatment when respectively normalized to the most stable and least stable reference genes (Figure 5). When using ACTIN and TATA, which were the most stable reference genes, significant increases in FD expression were observed in D55 relative to D25 samples, whereas no significant differences were evident between these two time points when using unstable reference genes such as GAPDH and RPS6.

## Discussion

Appropriate reference gene selection is dependent on species- and development stage-specific variability. For example, EF1α and Arm have been identified as reliable targets for normalizing RT-qPCR data derived from Australian plague locusts under varying rearing density conditions (Chapuis et al., 2011), while GAPDH and UCCR have served as *Spodoptera litura* reference genes (Lu et al., 2013). Indeed, no one reference gene or set of reference genes is universally appropriate across insect species. Zhang et al. (2015) demonstrated that while RPS15 and RPL13 were the most stable reference genes when comparing a range of *Helicoverpa armigera* larval tissue types, RPL27 and EF were more appropriate when evaluating adult samples. Five studies have explored reference gene selection in *Bemisia tabaci*, yet these studies have yielded conflicting results (Li et al., 2013; Su et al., 2013; Collins et al., 2014; Liang et al., 2014; Dai et al., 2017).

To date, reliable reference genes have been identified for many Hymenoptera species (Table 3), including *Cotesia chilonis* (Li et al., 2019), *Apis mellifera* (Lourenço et al., 2008), *Lysiphlebia japonica* (Gao et al., 2017a), *Aphidius gifuensis* (Gao et al., 2017b), *Tamarixia radiate* (Guo et al., 2020), and *Solenopsis invicta* (Cheng et al., 2013). *A. japonicus* is an important natural predator of a range of agricultural pests, and further molecular analyses of this species may thus support further biocontrol efforts. However, no prior studies have selected or validated reference genes in this parasitic wasp species. As such, eight commonly utilized reference genes were herein analyzed for their stability under four different sets of experimental conditions using a panel of software programs.

**TABLE 3:** Summary of the stability rankings of reference genes from studies conducted in Hymenoptera.

Organism	Treatment	Most stable gene(s)	Least stable gene(s)	All genes tested	References
*Aphidius gifuensis*	Developmental stages	ACTB, RPL13, PPI	RPL29	DIMT, ACTB, RPL3, PPI, TBP, RPII3, 18SrRNA, RPS18, AK, EF1A, RPL27, RPL29	Gao et al. (2017a)
	Sex	RPS18, ACTB, RPL13	RPL29		
	Tissue	RPL13, PRII3, RPS18	RPL27		
	Diverse diets	RPL13, RPL27, ACTB	18SrRNA		
*Apis mellifera*	Tissues in head	RPS18, GAPDH	TBP, RPL32	RP49, RPL32, RPS18, TBP, TUB,GAPDH	Moon et al. (2018)
	Insecticides	RPL32, ACTIN	RP18S, DORS	ACTIN, TUB, GST, GAPDH, HMBS, RPL32, PL13a, RPS18, SDHA, TBP, EF1A, AK, CHS6, DORS, 18S	Wieczorek et al. (2020)
	Tissues and season	RPS5, RPS18, GAPDH	—	ACTB, EIF, EF1, RPN2, RPS5, RPS18, GAPDH	Jeon et al. (2020)
*Atta sexdens rubropilosa*	Developmental stages	RPL18, EF1A	GAPDH, ACTIN	ACTIN, EF1A, EF1B, GAPDH, RPL18	Moreira et al. (2017)
	Tissue	RPL18, EF1B	EF1A, ACTIN		
*Bombus terrestris*	Tissue	PLA2, AK	TUB, GAPDH	AK, EF1A, ACTB, RPL13, PLA2, GAPDH, RPP2, TUB	Horňáková et al. (2010)
*Bombus lucorum*	Tissue	EF1A, PLA2	GAPDH		
*Cotesia chilonis*	Developmental temperature to low temperature	18S, H3, AK	—	18S, EF1, GAPDH, RPL10, RPL17, H3, AK, ACTB	Li et al. (2019)
	Developmental temperature to high temperature	GAPDH, ACTB	—		
	Cold temperature to low temperature	RPL17, RPL10	—		
	Cold temperature to high temperature	18S, H3, ACTB	—		
*Lysiphlebia japonica*	Developmental stages	DIMT, 18S, RPL13	RPS18,ACTB	EF1A, RPL13, PPI, RPII3, TBP, AK, TUB, 18S, ACTB, RPL27, RPS18, DIMT	Gao et al. (2017b)
	Sex	AK, RPL13, TBP	PPI, RPS18		
	Tissue	EF1A, PPI, RPL27	RPS18,TUB		
	Different diets	EF1A, RPL13, PPI	DIMT, RPS18		
*Monomorium pharaonis*	—	EF1A, GAPDH, TBP, TBLg2, HSP67	18S	EF1A, ACT5C, TBLb1, TBLg2, RPL5, RPS23, NADH, TBP, GAPDH, HSP83, 18S, HSP67	Ding et al. (2022)
*Solenopsis invicta*	—	RPL18, EF1B	ACTIN	RPL18, EF1B, ACTIN, TBP, GAPDH	Cheng et al. (2013)

Ribosomal proteins are highly conserved across different species (Petibon et al., 2021), and many ribosomal genes have successfully been leveraged as stable reference genes when conducting transcriptomic analyses (Liu et al., 2016; Gao et al., 2017a). Consistently, RPS3a and RPL13 were herein identified as the most stable *A. japonicus* reference genes across all conditions. In a prior study of *Agasicles hygrophila*o, these two genes were similarly found to exhibit stable expression in most tested experimental samples (Wang et al., 2019).In the present study, RPS6 was the second most stable reference gene across different tissue types and developmental stages in *A. japonicus,* while RPL13 was the most effective reference target when analyzing samples from different developmental stages or adults of different sexes. In *Rhopalosiphum padi*, RPS18 and RPL13 have been reported as the most stable reference genes across tissue types, while RPS6 was the second most stable such reference gene in response to temperature or antibiotic treatment conditions (Li et al., 2021).

ACTIN is an integral component of the cytoskeleton, controlling the structural integrity of cells such that it can be readily leveraged as a reference gene (Ponton et al., 2011). Indeed, ACTIN was found to be the most stable tested reference gene in *A. japonicus* under different tested diapause treatment conditions and in various tissues, in line with prior evidence regarding insecticide stress conditions in *Locusta migratoria* (Yang et al., 2014) and *Spodoptera litura* (Lu et al., 2013). Similarly, ACTIN showed high stability in other insects under different experimental sets, for the *Orchesella cincta*, ACTIN was the dominant most stable gene under temperature, desiccation and starvation treatments (de Boer et al., 2009), while high levels of ACTIN stability were evident in *Bradysia odoriphaga* specimens exposed to insecticides including chlorfluazuron or chlorpyrifos, in addition to remaining stable across developmental stages and tissue types (Fu et al., 2022). However, these results are not universal, with ACTIN having been established as the least stable gene in *Henosepilachna vigintioctopunctata* across different tissues, host plants, and stages of development (Lü et al., 2018). These findings emphasize the importance of individually evaluating reference gene stability under experimental conditions of interest.

Here reference gene stability was tested with the geNorm, NormFinder, BestKeeper, and ΔCt programs, and these different software applications each yielded differing results with respect to target gene stability. Under the developmental stages, the analysis results of NormFinder, geNorm and ΔCt are relatively similar, and the analysis results of BestKeeper are quite different from the other three analysis results. For example, RPS6/RPL13 ranks as the most stable reference gene in NormFinder, geNorm and ΔCt, but ranks 4th and 3rd in BestKeeper, respectively. Inconsistent results were obtained for these four software programs across tissue types, with RPS3a/RPL13, EF1α/Actin, TUB, and ACTIN ultimately being the most stable reference genes. When comparing specimens of different sexes, NormFinder and geNorm yielded similar results with EF1α and RPL13 as the two top-ranked genes, whereas TATA, Actin, and GAPDH were the bottom-ranked genes. In contrast, BestKeeper identified Actin and GAPDH as the two top-ranked genes. Under the tested diapause conditions, TATA and ACTIN were the top-ranked reference genes according to geNorm, NormFinder, and ΔCt, with Actin and TATA respectively being ranked first in the NormFinder and were TATA and ΔCt analyses. However, BestKeeper ranked RPL13 and EF1α as the two top genes. These inconsistent results across programs are attributable to the differences in the calculation strategies that they employ, with NormFinder and geNorm yielding more stable results for each reference gene. geNorm calculates M values for each reference gene to determine the optimal reference gene, with NormFinder using a similar approach while also analyzing each independent internal reference gene (Fu et al., 2013). BestKeeper produces correlation coefficients, standard deviations, and coefficients of variation for each gene pairing, enabling the simultaneous analysis of up to 10 genes (Chen and Lu, 2014). The inconsistent ranked lists produced by these programs can make the selection of optimal reference genes challenging. As such, RefFinder was used in this study owing to its ability to integrate these algorithms and to thereby better clarify overall candidate gene stability.

These results revealed marked differences in the optimal internal reference genes for use when comparing different sample types. This aligns well with growing interest in recent years to utilize more than one reference gene when normalizing gene expression data (Guo et al., 2021). The geNorm software offers the ability to establish the optimal number of reference genes for use through the calculation of pairwise variation (V_n/n + 1_) based on normalization factors NF_n_ and NF_n + 1_, where n is greater than or equal to 2. Using this algorithm, n is defined as the optimal number of reference genes when V_n/n + 1_ is under 0.15. Accordingly, the optimal number of reference gene combinations identified herein when comparing *A. japonicus* samples corresponding to different development stages, tissue types, sexes, and diapause treatments were RPL13 and RPS6, ACTIN and RPS6, RPL13 and EF1α, and ACTIN and TATA, respectively.

## Conclusion

In conclusion, these results underscore the importance of selecting application-specific reference genes when conducting RT-qPCR analyses of *A. japonicus,* as no one reference gene is optimal under all conditions. In this study, the most stable sets of reference genes were compared, leading to the identification of a combination of RPS3a and RPL13 as the most appropriate reference genes on average across the tested experimental conditions. However, the ideal reference genes ultimately varied among experiments, with RPL13 and RPS6 being the most reliable for developmental stage-based analyses, ACTIN and RPS6 as the most reliable when comparing different tissue types, ACTIN and TATA as the most stable when comparing different diapause treatments, and RPL13 and EF1α as the most stable when analyzing female and male *A. japonicus* specimens (de Boer et al., 2009; Chen and Lu, 2014; Lü et al., 2018; Wang et al., 2020).

## Data Availability

The raw data supporting the conclusion of this article will be made available by the authors, without undue reservation.
